# All-trans retinoic acid modulates pigmentation, neuroretinal maturation, and corneal transparency in human multiocular organoids

**DOI:** 10.1186/s13287-022-03053-1

**Published:** 2022-07-28

**Authors:** Helena Isla-Magrané, Maddalen Zufiaurre-Seijo, José García-Arumí, Anna Duarri

**Affiliations:** 1 Ophthalmology Research Group, Vall d’Hebron Institut de Recerca (VHIR), Vall d’Hebron Barcelona Hospital Campus, Passeig Vall d’Hebron 119-129, 08035 Barcelona, Spain; 2 Department of Ophthalmology, Vall d’Hebron Hospital Universitari, Vall d’Hebron Barcelona Hospital Campus, Barcelona, Spain; 3 Department of Surgery, Universitat Autònoma de Barcelona, Bellaterra, Spain

**Keywords:** All-trans retinoic acid, Ocular organoids, Stem cells, Retina, Cornea, Retinal pigment epithelium

## Abstract

**Background:**

All-trans retinoic acid (ATRA) plays an essential role during human eye development, being temporally and spatially adjusted to create gradient concentrations that guide embryonic anterior and posterior axis formation of the eye. Perturbations in ATRA signaling can result in severe ocular developmental diseases. Although it is known that ATRA is essential for correct eye formation, how ATRA influences the different ocular tissues during the embryonic development of the human eye is still not well studied. Here, we investigated the effects of ATRA on the differentiation and the maturation of human ocular tissues using an in vitro model of human-induced pluripotent stem cells-derived multiocular organoids.

**Methods:**

Multiocular organoids, consisting of the retina, retinal pigment epithelium (RPE), and cornea, were cultured in a medium containing low (500 nM) or high (10 µM) ATRA concentrations for 60 or 90 days. Furthermore, retinal organoids were cultured with taurine and T3 to further study photoreceptor modulation during maturation. Histology, immunochemistry, qPCR, and western blot were used to study gene and protein differential expression between groups.

**Results:**

High ATRA levels promote the transparency of corneal organoids and the neuroretinal development in retinal organoids. However, the same high ATRA levels decreased the pigmentation levels of RPE organoids and, in long-term cultures, inhibited the maturation of photoreceptors. By contrast, low ATRA levels enhanced the pigmentation of RPE organoids, induced the opacity of corneal organoids—due to an increase in collagen type IV in the stroma— and allowed the maturation of photoreceptors in retinal organoids. Moreover, T3 promoted rod photoreceptor maturation, whereas taurine promoted red/green cone photoreceptors.

**Conclusion:**

ATRA can modulate corneal epithelial integrity and transparency, photoreceptor development and maturation, and the pigmentation of RPE cells in a dose-dependent manner. These experiments revealed the high relevance of ATRA during ocular tissue development and its use as a potential new strategy to better modulate the development and maturation of ocular tissue through temporal and spatial control of ATRA signaling.

**Supplementary Information:**

The online version contains supplementary material available at 10.1186/s13287-022-03053-1.

## Introduction

All-trans retinoic acid (ATRA) is a metabolite of vitamin A (retinoids) that mediates functions required for eye growth and development during embryogenesis and vision [1]. ATRA functions as a morphogen signaling molecule that guides embryonic anterior/posterior axis formation of the eye during ocular development. The temporal and spatial regulation of ATRA creates a gradient of concentrations along the axes allowing the correct development and maturation of the different ocular structures, which are essential for the normal formation of the optic vesicle, optic cup, and anterior segment of the eye [2]. ATRA signaling is mainly divided into two distinct phases required for eye development: (i) in an early stage for optic cup formation, in which ATRA induces the separation of the neural retina, and retinal pigment epithelium (RPE) folded around the lens vesicle, and (ii) in a later stage for anterior eye formation, in which ATRA expression is critical for the completion of corneal, conjunctiva and lens formation [3]. In consequence, mutations in genes involved in ATRA signaling pathways lead to several ocular diseases such as microphthalmia, anophthalmia, coloboma, lens abnormalities, thicker corneas, thicker retinas, retinal dysplasia, and Mathew-Wood Syndrome, which represent a significant cause of childhood blindness or vision impairment [2]. At the intracellular level, the action of ATRA is through the binding to the nuclear receptor (RARs), which forms heterodimers with (RXRs). The RAR/RXR heterodimers activate target gene transcription via promoter RAR elements (RARE), inhibiting cell proliferation and promoting cell differentiation. Alternatively, ATRA binds peroxisome proliferator-activated receptors (PPAR), which heterodimerize with RXR. This complex binds its response elements (PPRE) implicated in lipid metabolism, cellular proliferation, and inflammatory responses. In parallel, to regulate the ATRA signaling, the excess intracellular levels of ATRA are degraded by cytochrome P450 enzymes (CYP26) to inactive forms [4].

There are discrepant results regarding ATRA effects on ocular tissue differentiation, maturation, and pigmentation of cells, leaving its precise contribution unclear. Most studies addressing the role of retinoids as signaling molecules in eye development have been done in animal models or diets deficient in vitamin A [5]. Despite these studies, limited data are available concerning the effect of ATRA on human eye development due to ethical concerns. Only a few studies using fetal and neonatal tissues provided unique insights and demonstrated that ATRA could influence diverse cellular aspects of early and late human ocular development. The ocular organoids are highly valuable to studying the eye as ocular organoid morphogenesis closely resembles human eye development, filling the gap between fetal tissue and animal models. We previously developed a 3D multiocular organoids derived from human induced pluripotent stem cells (hiPSC) in vitro consisting of retinal, retinal pigment epithelial (RPE), and corneal organoids [6]. Here, we use the human multiocular organoid model as a model system to study the role of ATRA in the in vitro differentiation and maturation of human ocular cells in the early stages of development.

## Methods

### Human-induced pluripotent stem cell culture

Human iPSC (CBiPS30-4F-5 line) was cultured and expanded in Matrigel-coated (Corning) plates in mTeSR1 medium (StemCell Technologies). Cells were passaged with 1 mM EDTA at a ratio of 1:3–1:6 every 5–7 days, and the medium changed daily. Cells were incubated at 37ºC in a humidified atmosphere and 5% CO_2_.

### Differentiation of hiPSC into ocular organoids

The differentiation protocol toward ocular (retinal, corneal, and RPE) organoids was previously described [6]. Briefly, hiPSC was cultured and differentiated into multi-zone ocular progenitor cells in Matrigel-coated plates with ocular medium (OM) consisting of Dulbecco’s Modified Eagle´s Medium/Nutrient Mixture F-12 (DMEM/F12), 5% fetal bovine serum, 0.1 mM non-essential amino acids, 2 mM GlutaMax, 1% N2, 1% B27 (all the previous reagents were from Gibco, Thermo Fisher Scientific), 10 mM β‐glycerolphosphate (Sigma-Aldrich), 10 mM nicotinamide (Sigma-Aldrich), and recombinant human IGF1 (10 ng/ml) (R&D Systems), supplemented with noggin (10 ng/ml) (Peprotech), DKK1 (10 ng/ml) (Sigma-Aldrich), and bFGF (10 ng/ml) (Peprotech) for 30 days. At this point, the different ocular structures were manually lifted and cultured in suspension to form ocular organoids. Organoids were kept in low attachment plates in OM medium with low ATRA (500 nM) or high ATRA (10 µM) (Sigma-Aldrich) concentrations [7] from days 30 to 90 to study the effect of ATRA on the maturation of ocular cells. In parallel, the cornea, neuroretina, and RPE regions were dissected during this process to obtain individual organoids as described [6]. Individual organoids were used for quantification (Table 1; Additional file 1: Fig. S1B) and characterization (retinal organoids in Fig. 3 and corneal organoids in Fig. 4). Alternatively, to study the maturation of photoreceptors, in particular, OM medium was supplemented with high ATRA (10 µM), triiodothyronine (T3; 20 nM) (Sigma-Aldrich), and/or taurine (100 µM) (Sigma-Aldrich) separately or in a combination of these three factors from days 90 to 120 of the maturation stage.

**Table 1:** Quantification (in %) of different organoids and organoids-containing pigmented areas cultured with high or low ATRA at day 90

Type of organoids	High ATRA			Low ATRA		
	% of organoids	% of pigmentated areas	n	% of organoids	% of pigmentated areas	n
Retina	57 ± 4	30 ± 6	115	56 ± 6	70 ± 3*	87
Cornea	40 ± 4	42 ± 4	81	12 ± 3*	76 ± 1*	18
RPE	3 ± 1	100	6	32 ± 2*	100	51

Data presented as mean ± SD (quantification of 207 organoids (high ATRA) and 160 organoids (low ATRA) in 4 replicates). Values indicated with stars are significantly different from those in high ATRA by Student’s t test; *p < 0.05. n; the number of organoids

### Histology and immunochemistry

Histology and immunohistochemistry were performed as described [6]. Primary antibodies are listed in Additional file 1: Table S1.

### Polymerase chain reaction and real-time quantitative PCR

Total mRNA extraction, cDNA synthesis, and PCRs were performed as described [8]. Primer sequences are listed in Additional file 1: Table S2.

### Western blot

Western blot analysis was performed as described [9]. Primary antibodies are listed in Additional file 1: Table S1 and gels are shown in Additional file 1: Fig. S4.

### Statistics

All quantitative data were analyzed using GraphPad Prism software (GraphPad Software). The unpaired two-tailed Student’s t test was applied to determine statistical significance between the two groups. Two-way ANOVA with post hoc test was used for more than two groups. Statistical significance was considered at < 0.05 with a confidence interval of 95%.

## Results

### ATRA inhibits pigmentation of ocular organoids

**Fig. 1:**
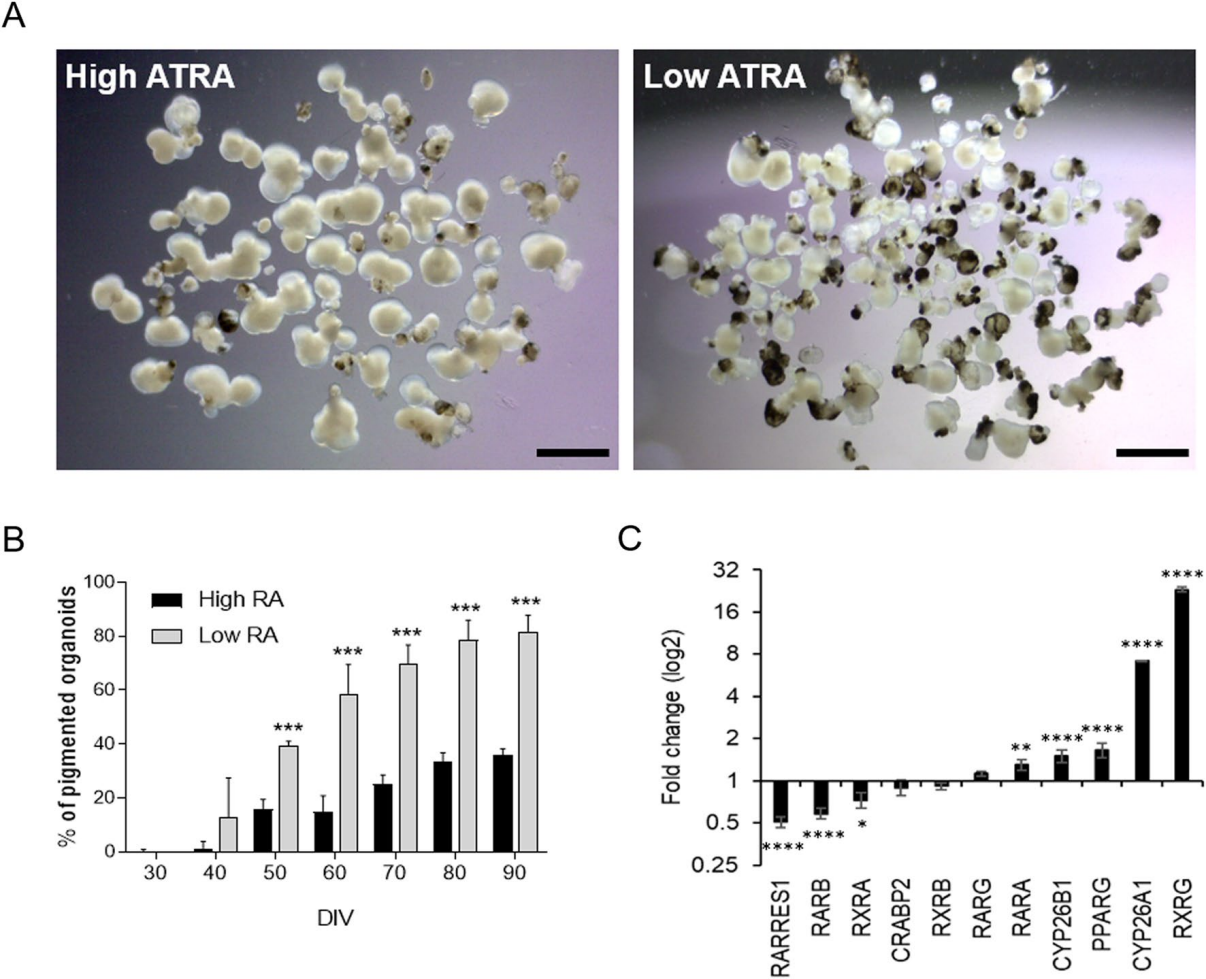
All-trans retinoic acid modulates pigmentation of hiPSC-derived multiocular organoids. **A** Representative images of multiocular organoids cultured in suspension in low or high all-trans retinoic acid (ATRA) concentrations at day 90. Scale bars: 3 mm. **B** Quantification of the number of pigmented ocular organoids at several days in vitro (DIV) is shown as mean ± SD (starting with *n* = 420 organoids (high ATRA) and *n* = 512 organoids (low ATRA) divided in 6 replicates). Asterisks represent statistical significance from a Student’s t test (***p < 0.0001). **C** Quantitative PCR analysis of ATRA signaling pathway components in multiocular organoids. Values are normalized to *GAPDH* and expressed as 2^−ddCt^ (log2 scale)*.* Data presented as mean ± SD (*n* = 6–10 organoids, three replicates). Values indicated by stars are significantly different from those in low ATRA (two-way ANOVA with Sidak’s multiple comparison test; *p < 0.05; **p < 0.001; ****p < 0.0001)

To test the effect of ATRA on ocular development, we treated multilocular organoids that consisted of a mix of retinal, retinal pigment epithelial (RPE), and corneal organoids [5] in high and low ATRA concentrations. Multiocular organoids cultured in low ATRA displayed higher levels of pigmentation, reaching 80% of total organoids at day 90. In contrast, high ATRA only presented pigmented areas in 40% of total organoids (Fig. 1A, B; Additional file 1: Fig. S1A). In parallel, we also observed that the ratio of the different ocular organoids varied depending on the concentration of ATRA medium supplementation. During the differentiation process with ATRA, individual organoids were obtained and quantified on day 90. High ATRA enhanced mainly retinal and corneal organoids with low levels of pigmentation, and only 3% ± 1 corresponded to RPE organoids (Table 1; Additional file 1: Fig. S1B). In opposition, low ATRA increased the number of pigmented areas/organoids, while the number of corneal organoids decreased and the number of pigmented RPE organoids increased (Table 1; Additional file 1: Fig. S1B). These results suggested that high ATRA long-term culture inhibited pigmentation of organoids and promoted corneal organoid formation. In contrast, low ATRA levels promoted epithelial pigmentation and reduced corneal tissue.

### High ATRA activates the expression of RA pathway genes

To explore the mechanisms underlying ATRA involvement in ocular differentiation, we examined the signaling pathway by which the ATRA functions in ocular progenitor cells. Multiocular organoids cultured in high ATRA concentrations expressed all signaling factors tested (Additional file 1: Fig. S2A-B). However, compared to the low concentration, high ATRA activated *RARA, RARG, RXRG*, the alternative RA receptor *PPARG*, and the degradation enzymes *CYP26* family (Fig. 1C). These data indicate that ATRA can activate both receptors in the multiocular organoid differentiation process, and the elevated ATRA concentration activates the ATRA catabolism.

### ATRA promotes neural retinal fate

**Fig. 2:**
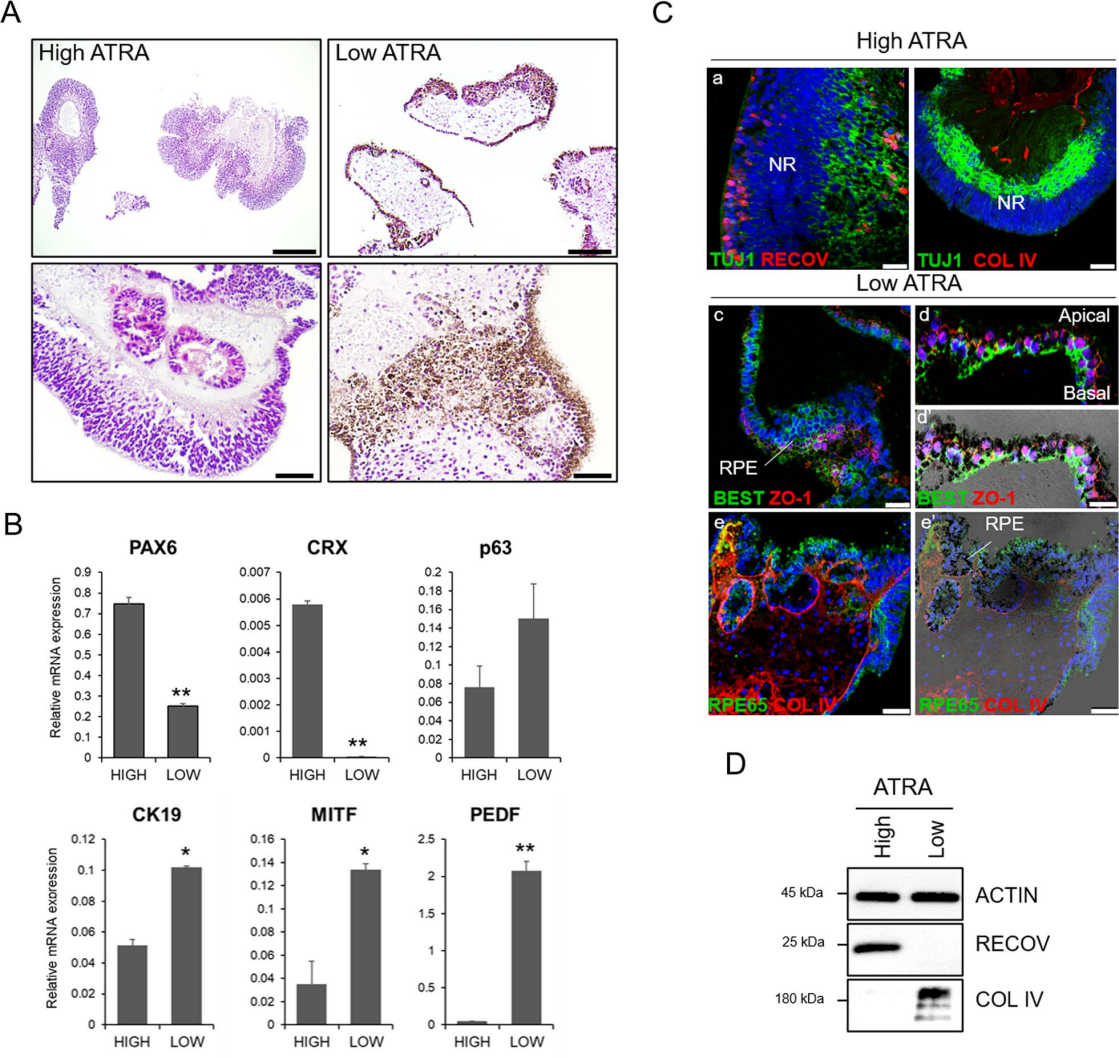
High ATRA promotes neuroretinal organoids, while low ATRA induces pigmented epithelial organoids. **A** Hematoxylin and eosin staining of paraffin sections from the multiocular organoids showing predominant neuroretinal (NR) organoids in high ATRA condition and pigment epithelial (PE) organoids in low ATRA condition at day 90. Scale bars: 250 µm (top images); 50 µm (bottom images). **B** Gene transcripts analysis by quantitative PCR in multiocular organoids. Values are normalized to *GAPDH*. Data presented as mean ± SD (*n* = 6–10 organoids, 3 replicates). Values indicated with stars are significantly different from those in high ATRA conditions (Student’s *t test*; *p < 0.01; **p < 0.001). **C** Confocal (a–e) and bright-field merged (e’) images of multiocular organoid paraffin sections stained with neuroretinal markers recoverin (RECOV) and TUJ1, and RPE markers bestrophin 1 (BEST), *zonula* *occludens* 1 (ZO1) and RPE65, and stromal collagen type IV (COL IV)). Nuclei are stained with DAPI. Scale bars: 50 µm in b,e,e’; 25 µm in a,c; 10 µm in d,d’. **D** Western blot analysis of RECOV and COL IV protein expression. Actin was used as the loading control

In vitro, ATRA regulates progenitors toward the photoreceptor cell fate and differentiation and survival of photoreceptors in the initial phase of retinal differentiation [8]. Similarly, we observed that ocular organoids from high ATRA cultures were mostly laminated neuroretina (NR) (Fig. 2A; Additional file 1: Fig. S1C) that expressed mainly retinal markers *PAX6, CRX*, and *recoverin* (Fig. 2B, C-a). NR displayed organized recoverin and TUJ1 positive cells resembling the outer nuclear layer and the ganglion cell layer, respectively, and maintained low collagen type IV deposition (Fig. 2C-a,b, D). To a lesser extent, corneal organoids present in the high ATRA culture expressed higher corneal marker *CK12* but lower levels of epithelial conjunctival marker *CK19 and p63* (Fig. 2B; Additional file 1: Fig. S2C), suggesting that corneal organoids shifted toward the conjunctival epithelium. In contrast, most pigmented organoids that emerged from low ATRA cultures showed an epithelium resembling the native RPE with dense stroma (Fig. 2A; Additional file 1: Fig. S1C), corroborated through the expression of RPE genes *MITF, PEDF, SIL,* and *TYR* (Fig. 2B, Additional file 1: Fig. S2C ) and RPE markers bestrophin-1, ZO-1 and RPE65 (Fig. 2C-c-e). RPE monolayer displayed apical-basolateral polarization at the organoid surface, as shown by tight junctions on the apical side and bestrophin-1 at the basolateral site (Fig. 2C-d,d’). Moreover, RPE cells also secreted collagen IV forming a basal Bruch’s-like membrane (Fig. 2C-e,e', D), a key feature of native RPE cells. These findings indicate that high ATRA concentrations promoted neuroretinal and corneal epithelium formation, whereas lower ATRA levels promoted epithelium formation (RPE and conjunctiva) and pigmentation.

### ATRA inhibits the maturation of photoreceptors

**Fig. 3:**
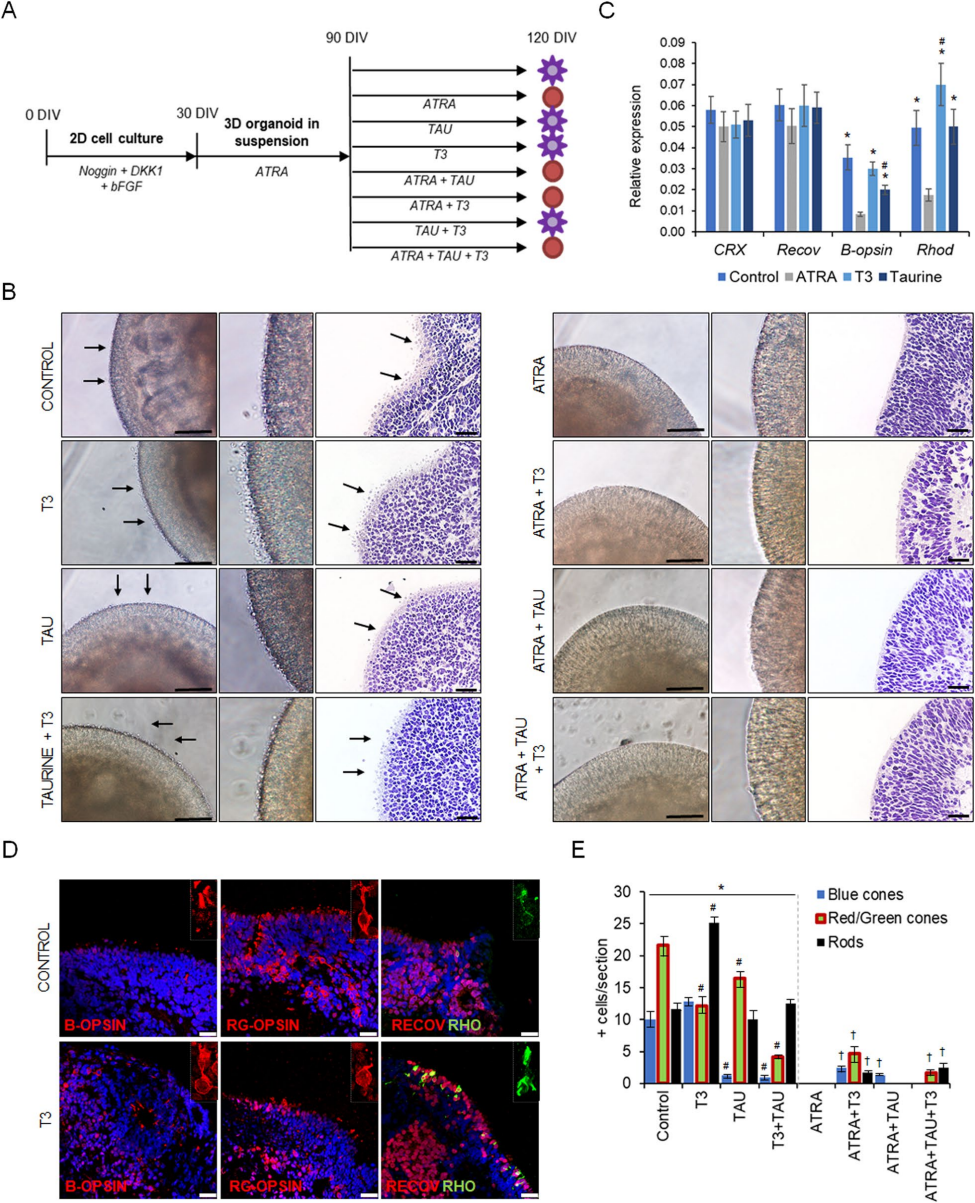
Specific addition of all-trans retinoic acid (ATRA), triiodothyronine (T3), and taurine (TAU) modulates the generation and maturation of cone and rod photoreceptors in retinal organoids. **A** Diagram of the experimental design, showing the addition of stage-specific factors during retinal organoid differentiation in 3 phases as follows: (i) noggin, DKK1, and bFGF from day 0 to 30; (ii) ATRA (10 µM) from days 30 to 90; and (iii) medium (control), ATRA, T3 and TAU individually and in combination with each other, from days 90 to 120. Purple suns indicate retinal organoids with developed inner and outer segments (IS/OS) of photoreceptors, and red circles indicate retinal organoids without developed IS/OS. **B** Bright field and hematoxylin and eosin staining of retinal organoids treated with medium (control), ATRA, T3, and TAU; and a combination. Middle panels show magnification of IS/OS of photoreceptors. Arrows indicate the developing IS/OS. Scale bars: 50 µm. **C** Quantitative PCR of gene expression in retinal organoids at day 120. Values are normalized to *GAPDH*. Data presented as mean ± SD (*n* = 3). Statistical significance was calculated by two-way ANOVA followed by Tukey’s multiple comparison tests (*p* < 0.05) comparing with ATRA (*); comparing control, T3 and taurine groups (#). **D** Representative immunostaining images of retinal organoids treated with medium (control) or T3, stained with photoreceptor-specific markers recoverin (RECOV), blue-opsin (B-opsin), red/green-opsin (RG-opsin), and rhodopsin (RHO). Nuclei are stained with DAPI. Scale bars: 25 µm. **E** Quantification of number of positive cells expressing blue-opsin (Blue cones), red/green-opsin (red/green cones) or rhodopsin (Rods) in retinal organoid paraffin sections (*N* = 3 different experiments, *n* = 5–7 retinal organoids). Two-way ANOVA calculated statistical significance (*p* < 0.05) comparing the non-ATRA group with ATRA data (*); comparing TAU, T3, and TAU + T3 with Control (#); and comparing ATRA with the ATRA + supplement groups (†)

As we have shown, ATRA promotes NR differentiation until day 90. To assess how ATRA affects photoreceptor maturation in long-term cultures and increases the generation of cone and rod photoreceptors in retinal organoids, we studied the optimal addition of ATRA, taurine, and triiodothyronine (T3) or a combination of them in the medium from day 90 to 120 of differentiation (Fig. 3A). These supplements have been shown to promote cone and rod photoreceptor formation [10]. We observed that short-term treatment with ATRA from days 30 to 90, followed by medium alone or medium supplemented with T3 or taurine from days 90 to 120, increased the production of cone/rod photoreceptors (Fig. 3). The translucent projections, representing likely photoreceptor inner segments, connecting cilia, and developing outer segments at the apical site of the retinal organoids, were visible on day 120 (Fig. 3B). Using immunolabeling and qPCR, we observed that the medium promoted the emergence of the red/green (RG)-cone photoreceptor, whereas T3 promoted an equal number of blue (B)-cone and RG-opsin photoreceptors and a higher number of rods compared to all other conditions (Fig. 3C–E). Taurine blocked the B-cone emergence while maintaining similar levels of RG-cone and rod photoreceptors (Fig. 3C–E). Insistingly, the simultaneous addition of T3 and taurine decreased the number of cone photoreceptors. In contrast, long-term ATRA culturing suppressed the overall number of mature photoreceptors in all conditions. Only ATRA + T3 supplementation slightly increased the emergence of all photoreceptor types, although in less proportion than T3 alone (Fig. 3E). Despite these differences, the levels of recoverin and CRX were similar among groups (Fig. 3C), indicating proper photoreceptor fate specification in organoids. Our data suggest that long-term high ATRA supplementation inhibited photoreceptor maturation but not photoreceptor fate and number. In addition, T3 was the best supplement to enhance the maturation of rod photoreceptors, while medium without any supplement promoted the emergence of cone photoreceptors.

### ATRA maintains corneal organoids transparency

**Fig. 4:**
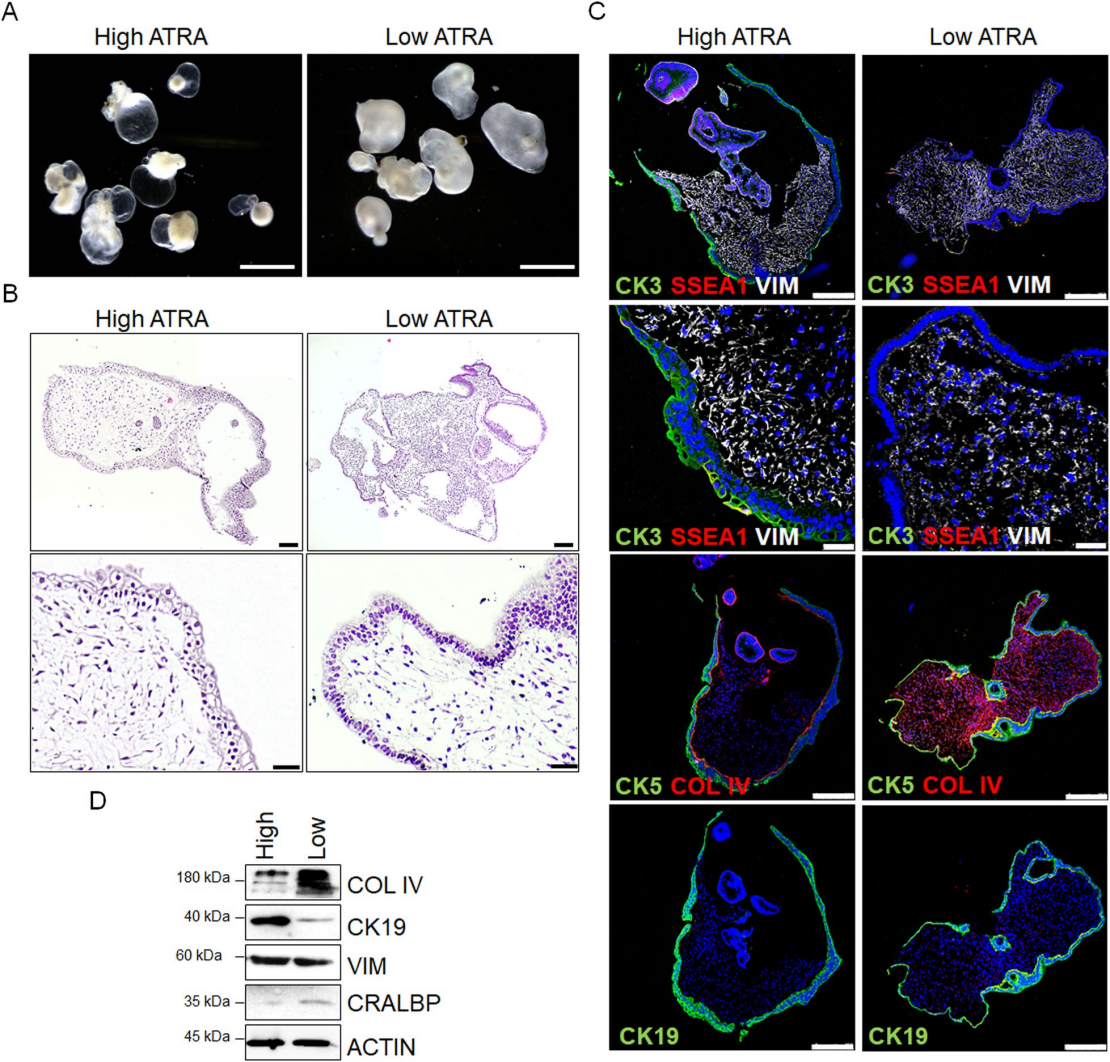
High ATRA concentration increases corneal organoids transparency and decreases collagen deposition in the stroma. **A** Representative images of corneal organoids at day 120 cultured in high ATRA (transparent corneal organoids) and low ATRA (opaque corneal organoids) concentrations. Scale bars: 3 mm. **B** Hematoxylin and eosin staining of paraffin sections of transparent and opaque corneal organoids. Scale bars: 150 µm (upper panels); 50 µm (lower panels). **C** Immunofluorescent images of transparent (high ATRA) and opaque (low ATRA) corneal organoids expressing corneal markers CK3, corneal-conjunctival markers CK5 and MUC1, conjunctival marker CK19, and limbal stem cell marker SSEA1. Stromal cells are stained with vimentin (VIM) and collagen type IV (COL IV). Nuclei are stained with DAPI. Scale bars: 50 µm. **D** Western blot analysis of protein levels related to stroma (COL IV and VIM), cornea-conjunctiva (CK19), and RPE (cellular retinaldehyde–binding protein, CRALBP) in corneal organoids. Actin was used as a loading control

ATRA is essential for completing corneal, conjunctiva, and lens during eye development, and deficiency of vitamin A or mutations in ATRA pathways lead to the opacity of the cornea, among other defects [11]. The corneal epithelium consists of stratified squamous cells and stromal keratocytes that synthesize extracellular matrix proteins bringing transparency to the cornea. We studied the effect of ATRA on corneal organoids. High levels of ATRA during maturation induced corneal transparency, while low levels of ATRA triggered the opacity of corneal epithelium (Fig. 4A). Transparent organoids presented stratified squamous epithelium expressing CK3 in the apical site and conjunctival CK5 and CK19 (Fig. 4B-C). Some cells also expressed SSEA1 corresponding to potential limbal stem cells (Fig. 4C; Additional file 1: Fig. S3). By contrast, low ATRA-derived opaque organoids contained mainly stratified columnar epithelial cells on the surface (Fig. 4B), positive for CK5, CK19, and MUC1 but negative for CK3 (Fig. 4C; Additional file 1: Fig. S3). We also observed that high ATRA-derived transparent corneal organoids contained increased expression of vimentin + cells in the stroma but reduced collagen type IV deposition, confirmed by Western blot (Fig. 4D). Moreover, these organoids also expressed higher levels of CK19 corresponding to cornea-conjunctiva and lower levels of CRALBP, an RPE marker (Fig. 4D). These results suggest that ATRA has a relevant biological role in maintaining stromal integrity and homeostasis in cornea and conjunctiva formation during tissue differentiation.

## Discussion

The study performed here enables us to present multiocular organoids as a new model to study the action of ATRA during eye development. Our results display a direct correlation between the presence of ATRA and the pigmentation of different ocular organoids, the maturation of photoreceptors, and the transparency of the corneal organoids. The correlation observed was dependent on the time-window administration and ATRA concentration. This correlation is due to temporally and spatially restricted expression patterns of individual components of ATRA in mammals [11]. Multilocular differentiation specification toward retinal and corneal fates is accompanied by a shift in the balance between ATRA receptors, activating RARα, RARγ, and RXRγ, in addition to PPARγ and CYP26. This could be due to the elevated ATRA concentration. In contrast, retinal pigment epithelium was induced by the activation of RARβ and RXRα, the latter important for RPE survival [12], suggesting that ATRA’s biological functions depend on its concentrations and are broader than previously described.

In the initial stages of retinal organoid differentiation, retinal fate induction and the correct lamination of the neuroretina required ATRA. This is in line with mouse eye development, in which ATRA signaling is active in the optic cup, neuroretina, and the RPE in the early stages of development (E8.5–10.5) [11]. However, in later stages, contrary to Zerti et al. [10], we observed that ATRA from day 90 to 120 inhibited the maturation of all photoreceptor types, suggesting that long-term high ATRA exposure slows down photoreceptor maturation. Moreover, we also observed that in the absence of ATRA, the medium containing only IGF1 and FBS, or the presence of T3, enhanced the maturation of rod photoreceptors and both blue and green/red cone photoreceptors at similar levels, similarly to Sawant et al*.* [13]. Interestingly, on day 120, we obtained a high number of photoreceptors expressing opsins, specifically RG-opsin and rhodopsin, compared to other protocols that need longer culture times.

Likewise, corneal organoid development and transparency also required high ATRA during maturation, but in that case, in a more extended exposition. During mouse eye development, ATRA signaling is active in the second phase (E10.25) of the anterior segment formation that involves surface ectoderm and periocular mesenchyme, resulting in the formation of cornea and lens [2]. In the absence of ATRA, we observed that corneal organoids became opaque likely due to an incorrect mesenchymal cell differentiation underneath the corneal epithelium and the abnormal corneal stroma. The modulatory effect of ATRA has been shown to enhance proliferation and stratification of corneal keratocytes and reduce mobility at 1 µM [14]. By contrast, ATRA deprivation in the perioptic mesenchyme—derived from neural crest cells— is described to disrupt anterior segment morphogenesis due to increased perioptic mesenchyme growth and cause the replacement of normal epithelial by an abnormal keratinized stratified squamous epithelium [15]. These results coincide with previous data supporting that a decrease in the ATRA signaling pathway potentially induces human corneal opacity and other pathologies such as glaucoma, Axenfeldt/Rieger syndrome, or congenital anterior dysgenesis.

We also observed that organoids showed significantly less pigmentation and epithelial formation in high ATRA than with low ATRA concentration cultures. ATRA has been reported to regulate RPE-choroid barrier function by increasing the expression of tight junction proteins and barrier function [16, 17], inhibiting the proliferation and adhesion of retinal pigment epithelial [18], and enhancing pigmentation of epithelia in a concentration range of 1–100 nM and has a depigmentation capacity in a range of 5–20 µM [19]. In this line, the epithelial layer developed in low ATRA cultures closely resembles RPE, with polarized, pigmented epithelial cells and deposition of collagen type IV in the stroma. Moreover, the pigmentation levels correspond to the higher expression of specific enzymes, such as melanin-synthesizing enzymes TYR. We hypothesize that high ATRA stimulates neuroretinal lineage development and is detrimental to RPE lineage by i) inhibiting RPE formation and maturation; ii) upregulating the secretion (by the existing RPE) of PEDF, bFGF, EGF, or NGF that enhance the development and maturation of neuroretina [20]; and iii) decreasing the deposition of extracellular matrix [21].

## Conclusions

These results gain insights into how ATRA influences complex multiocular organoids' ocular tissue development. Therefore, underlying the importance of finding new strategies which allow ATRA distribution temporally and spatially during organoid generation. These studies have permitted a better understanding of embryonic eye development and ocular function.

## Supplementary Information

**Additional file 1**. “Supplementary Information” contains tables for antibodies and primers (Tables S1 and S2) and figures for the characterization of multiocular organoids and Western blots.

## Data Availability

Not applicable.

## Abbreviations

ATRA: All-trans retinoic acid
B-cone: Blue cone
bFGF: Basic fibroblast growth factor
CK3: Cytokeratin 3
CK5: Cytokeratin 5
CK12: Cytokeratin 12
CK19: Cytokeratin 19
COL IV: Collagen type IV
CRABP2: Retinoic acid binding proteins 2
CRALBP: Cellular retinaldehyde-binding protein
CRX: Cone-rod homeobox
CYP26: Cytochrome P450 family
DAPI: 4',6-Diamidino-2-phenylindol
DIV: Days in vitro
DKK-1: Dickkopf-1
GAPDH: Glyceraldehyde 3-phosphate dehydrogenase
hiPSC: Human induced pluripotent stem cells
IGF-1: Insulin growth factor 1
MITF: Melanocyte-inducing transcription factor
MUC1: Mucin 1
NR: Neuroretina
P63: Delta-N tumor protein p63
PAX6: Paired box 6
PEDF: Pigment epithelium-derived factor
PPAR: Peroxisome proliferator-activated receptor gamma
qPCR: Quantitative polymerase chain reaction
RAR: The retinoic acid receptor
RARE: Retinoic acid response elements
RARRES1: Retinoic acid receptor responder 1
RAX: Retinal homeobox protein Rx
RECOV: Recoverin
RG: Red/green
RHO: Rhodopsin
RPE: Retinal pigment epithelium
RPE65: Retinal pigment epithelium-specific 65 kDa protein
RXR: Retinoid X receptor
SD: Standard deviation
SIL: Silver
SSEA1: Stage-specific embryonic antigen 1
T3: Triiodothyronine
TAU: Taurine
TUJ1: Beta-III tubulin
TYR: Tyrosinase
ZO-1: *zonula occludens*
