# Supplementary material for: All-trans retinoic acid modulates pigmentation, neuroretinal maturation, and corneal transparency in human multiocular organoids
Source: Stem Cell Res Ther. 2022 Jul 28;13:376. doi: 10.1186/s13287-022-03053-1 (PMC9330659; doi:10.1186/s13287-022-03053-1)
Supplement: Supplementary file 1 — Additional file 1. “Supplementary Information” contains tables for antibodies and primers (Tables S1 and S2) and figures for the characterization of multiocular organoids and Western blots. [file 13287_2022_3053_MOESM1_ESM.pdf]

All-trans retinoic acid modulates pigmentation, neuroretinal maturation, and corneal transparency in human multiocular organoids.

Helena Isla-Magrané, Maddalen Zufiaurre, José García-Arumí, Anna Duarri*

Tables: 2

Figures: 4

Supplementary Table S1: List of primary antibodies

Antibody	Supplier and reference	Species	Dilution (IF/WB)
**Actin**	MP Biomedicals, 691001	Mouse	- / 1:1000
**Bestrophin**	Santa Cruz Biotechnology, sc-32792	Mouse	1:25
**B-Opsin**	Millipore, AB5407	Rabbit	1:100
**Collagen type IV**	Abcam, ab6586	Rabbit	1:100 / 1:1000
**CRALBP**	Santa Cruz Biotechnology, sc-28193	Rabbit	- / 1:500
**Cytokeratin 19**	Genetex, GTX112666	Rabbit	1:100 / 1:1000
**Cytokeratin 3/2p**	Santa Cruz Biotechnology, sc-80000	Mouse	1:100
**Cytokeratin 5**	Abcam, ab24647-50	Rabbit	1:100
**MUC1**	Genetex, GTX100459	Rabbit	1:100
**Recoverin**	Millipore, AB5585	Rabbit	1:500 / 1:5000
**RG-Opsin**	Millipore, AB5405	Rabbit	1:100
**Rhodopsin**	Sigma, O4886	Mouse	1:500
**RPE65**	Santa Cruz Biotechnology, sc-73616	Mouse	1:100
**SSEA1**	Iowa, MC-480	Mouse	1:2
**Tuj1**	Covance, MMS-435P	Mouse	1:1000
**Vimentin**	Cell signaling kit Arigobio, SQab1721	Rabbit	1:100 / 1:1000
**ZO-1**	Millipore, ab2272	Rabbit	1:100

Supplementary Table S2: List of primers used in PCR and qPCR

Gene	Forward	Reverse
CK12	AGCAGAATCGGAAGGACGCTG	ACCTCGCTCTTGCTGGACTGAAA
CK19	ACAGCCACTACTACACGACC	CCTGTTCCGTCTCAAACTTGGT
CRABP2	TCGGAAAACTTCGAGGAATTGC	CCTGTTTGATCTCCACTGCTG
CRX	TCCAGGGTTCAGGTTTGGTT	CATCTGTGGAGGGTCTTGGG
CYP26A1	CACCGTACGGGTGATGGGCG	GCTGGCCAGTGGACCGACAC
CYP26B1	ACCGGCCACTGGCTGCTG	ACGTTGATGGCCTCGGGGTG
GAPDH	CCTGCACCACCAACTGCTTAG	TGGCATGGACTGTGGTCATG
MITF	GTGCCAACTTCTTTCATCA	ACCTAAACCGTCCATTCA
OPN1SW	TAGCAGGTCTGGTTACAGGATG	GAGACGCCAATACCAATGGTC
P63	GAAAACAATGCCCAGACTCAATTT	TCTGCGCGTGGTCTGTGTTAT
PAX6	TCTAATCGAAGGGCCAAATG	TGTGAGGGCTGTGTCTGTTC
PEDF	AGATCTCAGCTGCAAGATTGCCCA	ATGAATGAACTCGGAGGTGAGGCT
PPARG	TACTGTCGGTTTCAGAAATGCC	GTCAGCGGACTCTGGATTCAG
RARA	GGGCAAATACACTACGAACAACA	CTCCACAGTCTTAATGATGCACT
RARB	TCCGAAAAGCTCACCAGGAAA	GGCCAGTTCACTGAATTTGTCC
RARG	ATGCTGCGTATCTGCACAAG	AGGCAAAGACAAGGTCTGTGA
RARRES1	AAACCCCTTGGAAATAGTCAGC	GGAAAGCCAAATCCCAGATGAG
RECOVERIN	TCTACGACGTGGACGGTAACG	CGTCCTCGGGAGTGATCATT
RHODOPSIN	GCTGGTCCAGGTACATCCCC	TGAAGACGAGCTGCCCATAG
RXRA	ATGGACACCAAACATTTCCTGC	GGGAGCTGATGACCGAGAAAG
RXRB	ACGGCTATGTGCAATCTGC	CGGATGGTGCGTTTGAAGAA
RXRG	CCGGATCTCTGGTTAAACACATC	GTCCTTCCTTATCGTCCTCTTGA
SIL	GTTGATGGCTGTGGTCCTTG	CAGTGACTGCTGCTATGTGG
TYR	ACTTACTCAGCCCAGCATC	GGTTTCCAGGATTACGCC

FIGURE S1

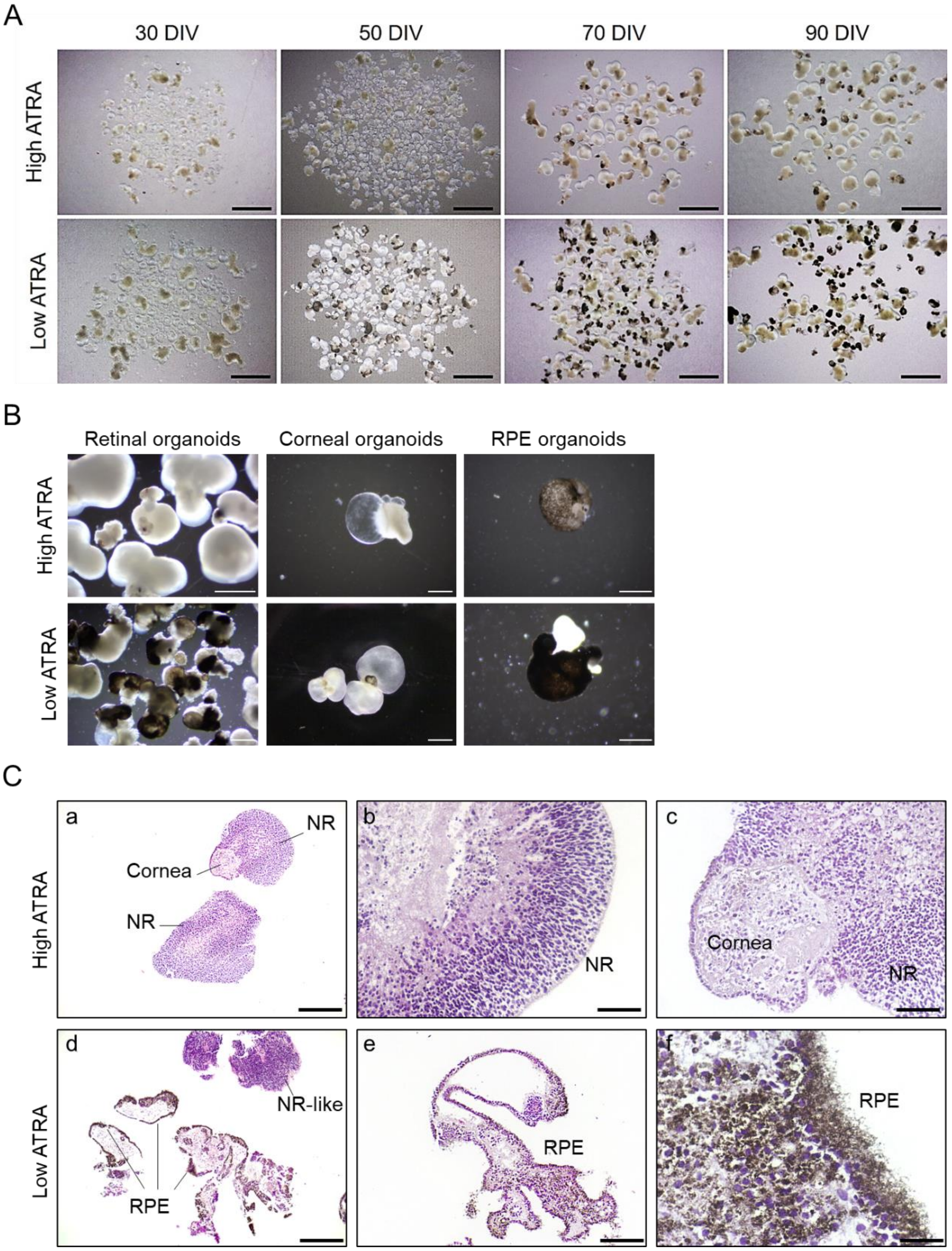

Figure S1: Low all-trans retinoic acid induces pigmentation in hiPSC-derived multiocular organoids. A) Representative images of multiocular organoids in suspension, cultured in low or high all-trans retinoic acid (ATRA) concentrations at 30, 50, 70, and 90 days *in vitro* (DIV). Scale bars: 3 mm. B) Representative images of individual organoids. Scale bars: 500 µm. C) Hematoxylin and eosin staining of paraffin sections showing a predominant neuroretinal (NR) and corneal organoids in high ATRA conditions and mostly retinal pigment epithelial (RPE) organoids in low ATRA conditions. Scale bars: 250 µm (a,d.e); 50 µm (b,c); 25 µm (f).

FIGURE S2

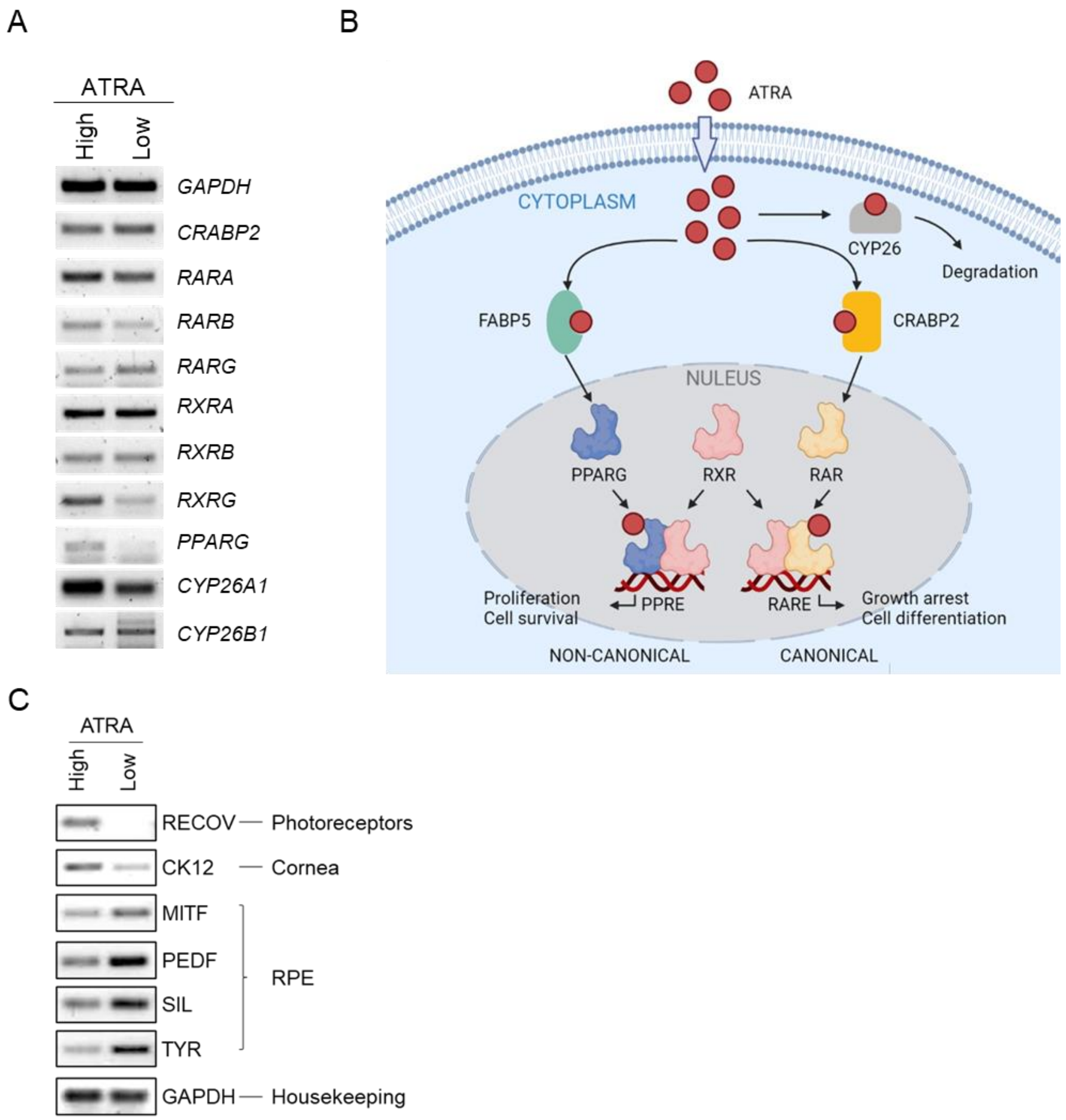

Figure S2: All-trans retinoic acid pathway. A) Analysis of mRNA expression by PCR of the indicated genes in multiocular organoids cultured in high and low ATRA concentrations. *GAPDH* levels are shown as a loading control. B) Scheme showing ATRA signaling pathway. ATRA is diffused inside the cytoplasm and transported into the nucleus by retinoic acid-binding proteins (CRABPs). Then, ATRA binds to the retinoic acid receptor (RAR) that in turn form heterodimers with retinoid X receptor (RXR), binding to retinoic acid response elements (RAREs) initiation of gene transcription. Alternatively, ATRA can bind the peroxisome proliferator-activated receptor gamma (PPAR). Activated receptors form heterodimers with RXR and bind to the PPAR response element (PPRE). The excess ATRA is degraded by the cytochrome P450 family (CYP26) proteins, which oxidize ATRA to various inactive metabolites. C) Gene transcripts analysis by PCR of ocular-specific regions. *GAPDH* was used as a housekeeping gene.

FIGURE S3

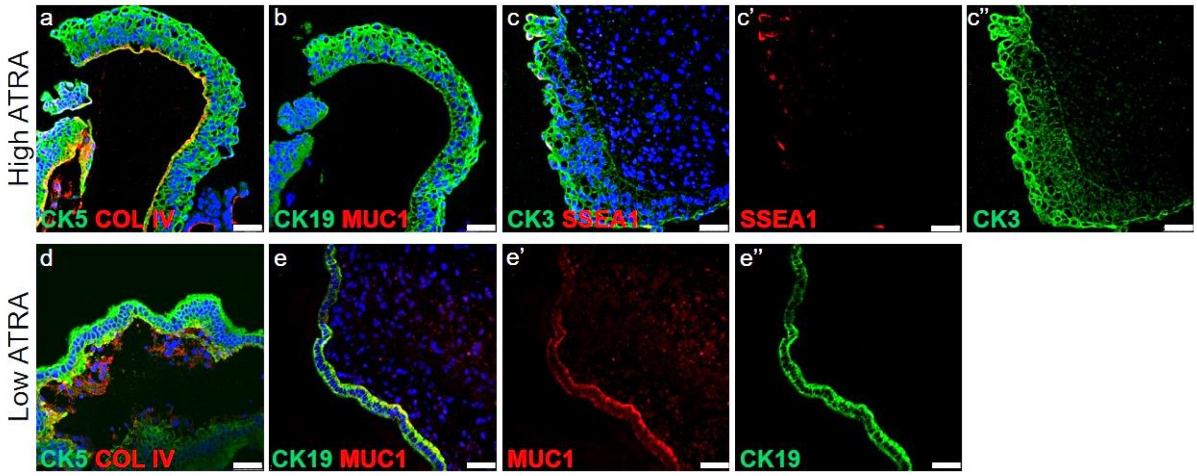

Figure S3: Corneal organoids treated with low and high ATRA concentrations. Immunofluorescent images of corneal organoid paraffin sections stained with CK3 (corneal marker), CK5 and CK19 (corneal-conjunctival marker), SSEA1 (limbal stem cell marker), and MUC1 (mucin 1, goblet cell marker). Scale bars: 50 µm.

FIGURE S4

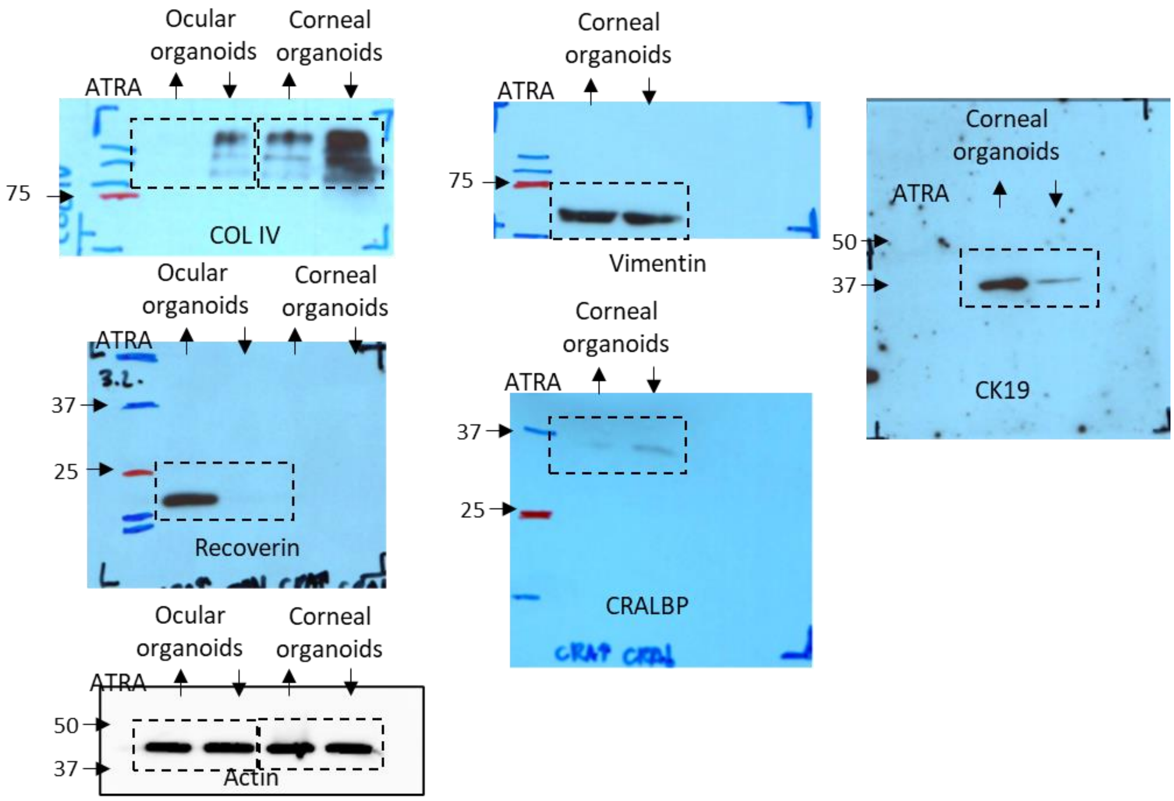

Figure S4: Full scans of Western blot data. Full scans of Western blot of Figure 2 (Collagen type IV, Recoverin, and actin) and Figure 4 (Collagen type IV, Vimentin, CRALBP, CK19, and actin). Due to the low protein concentration obtained from ocular and corneal organoids, Western blot membranes were cropped before the immunoblotting to maximize the antibodies used for each membrane. Actin was revealed using ImageQuant LAS 4000 (GE Healthcare). Dashed squares represent the images of bands shown in the corresponding figure.
